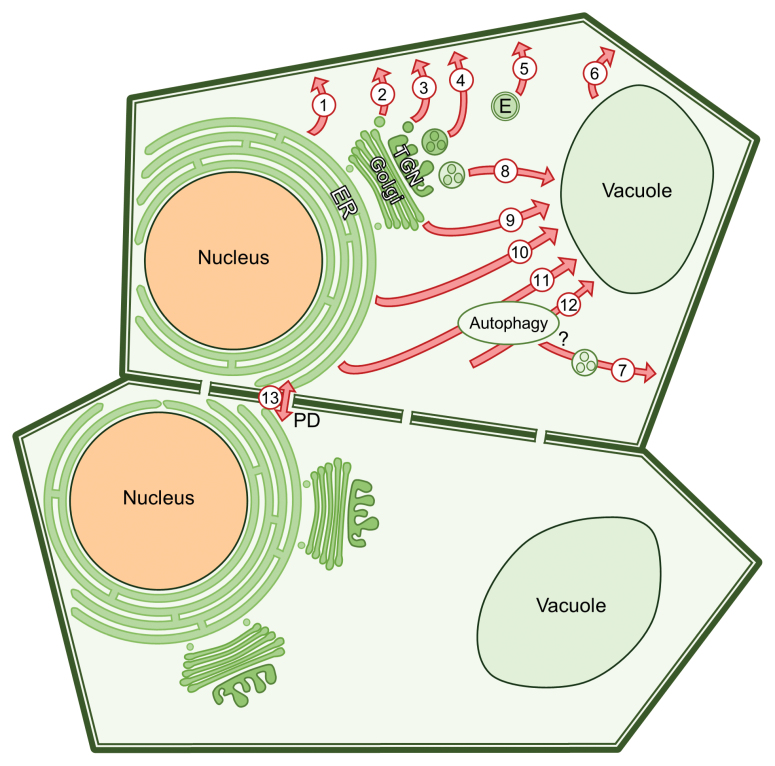# Protein and membrane trafficking routes in plants: conventional or unconventional?

**DOI:** 10.1093/jxb/erx435

**Published:** 2017-12-18

**Authors:** Daphne R Goring, Gian Pietro Di Sansebastiano

**Affiliations:** 1 Department of Cell & Systems Biology, University of Toronto, Canada; 2 DiSTeBA (Dipartimento di Scienze e Tecnologie Biologiche ed Ambientali), University of Salento, Italy

**Keywords:** Endoplasmic reticulum, Golgi, membrane-bound compartments, membrane trafficking, plasmodesmata, protein trafficking, trafficking, trafficking pathways, unconventional protein trafficking, vacuole

**The conventional trafficking of proteins, membranes and metabolites between membrane-bound compartments in the cell uses highly conserved pathways. However, this does not always account for the way some cargo are sorted to their final destination, and ongoing research is focused on defining these ‘unconventional’ routes and investigating their purpose. In this special issue, a collection of reviews discusses the current literature in this field, proposing thought-providing mechanistic models, and exploring where these unconventional trafficking pathways are employed, from basic cellular functions to plant–microbe interactions.**

All eukaryotic cells, including those of plants, are composed of a series of membrane-enclosed organelles, and sorting proteins, lipids and metabolites across these membranes to the correct compartments is a basic and essential cellular function. Several trafficking pathways have been well-characterized, but the increasing sensitivity of research tools and the use of new experimental systems has revealed that these known sorting mechanisms do not account for the way some proteins and metabolites end up in their final destination. Initially reported as anomalies, new unconventional trafficking routes are being defined and, furthermore, one could start to debate whether they would still be considered ‘unconventional’ in those contexts where they appear to be more prevalent than the conventional pathways. This topic was part of the lively discussions at the ‘Unconventional Protein and Membrane Traffic’ meeting held in Lecce, Italy in 2016, and form the basis for this special issue. The meeting brought together researchers working on a range of biological systems, including yeast, animals and plants, to present different viewpoints on these unconventional trafficking mechanisms (Pompa *et al.*, 2017). This special issue focuses on the plant perspective, with the reviews discussing differences and commonalities with the other eukaryotic trafficking systems.

## The endoplasmic reticulum as the trafficking start point

The conventional trafficking pathway starts at the endoplasmic reticulum (ER) and routes proteins to the Golgi apparatus (reviewed by Bellucci *et al.*, 2017; Wang *et al.*, 2017). These two compartments are closely associated with each other to facilitate movement of cargo between them. For example, the ER forms an extensive network throughout the plant cell, and the Golgi bodies are associated with ER in part through golgin-tethering complexes (Osterrieder *et al.*, 2017; see also the Insight article by Vitale and Pedrazzini, 2017). From the ER, these proteins are exported to the *cis*-side of the Golgi and are transported through the Golgi stacks where protein modifications such as glycosylation can occur. Proteins destined for the vacuole are then exported to the *trans*-Golgi network (TGN; also known as the early endosome) and then onto the prevacuolar compartment (also called the late endosome/multivesicular body). From there, these proteins are trafficked to the vacuole. Proteins destined for the cell surface can be sorted at the Golgi or TGN into transport vesicles for delivery to the plasma membrane.

Unconventional trafficking pathways are also initiated at the ER, and Bellucci *et al.* (2017) discuss a thought-provoking model that proposes the ER as the central hub for unconventional protein secretion (UPS). Part of this concept involves thinking about the orientation of organellar membranes relative to the cytoplasm and defining ‘external space’ for protein secretion beyond the cell surface to include the lumen of the secretory compartments as well as the inner space of endosymbiotic organelles (plastids and mitochondria). Examples of this type of unconventional protein secretion include an ER to plasma membrane route that bypasses the Golgi and vesicles, probably implemented through direct ER–plasma membrane contact sites. Similarly, there is also the possibility of direct transport from the ER to vacuole as discussed in more detail below.

## How many different routes for protein secretion?

To enter the conventional secretory pathway, proteins have signal peptides that are recognized for translocation into the ER during synthesis. Once these proteins are exported to the Golgi apparatus from the ER, there are two potential routes to the plasma membrane: (1) cargo are loaded into secretory vesicles at the Golgi or (2) cargo are exported to the TGN and then loaded into secretory vesicles for transport to the plasma membrane (reviewed by Wang *et al.*, 2017). However, there are also proteins that lack a signal peptide for translocation into the ER, yet are found secreted in the apoplast (reviewed by Rodriguez-Furlan *et al.*, 2017; Wang *et al.*, 2017). These leaderless secretory proteins (LSPs) are located in the cytosol, and one potential unconventional secretory mechanism could involve direct translocation across the plasma membrane. Another proposed mechanism suggests that these cytosolic LSPs are sequestered during the formation of exocyst-positive organelles (EXPOs), followed by EXPO fusion to the plasma membrane (Wang *et al.*, 2010; Wang *et al.*, 2017). Other unconventional secretory pathways have been shown to involve the direct fusion of a vacuole or an autophagy-related structure with the plasma membrane; for example, this might be to release defence compounds during pathogen attack (reviewed by Pecenkova *et al.*, 2017; Wang *et al.*, 2017). Regardless of the trafficking pathway, the final step of cargo delivery involves membrane fusion events which are facilitated by docking complexes. Pecenkova *et al.* (2017) review the literature on the exocyst docking complex, and discuss how specificity may in part be achieved by different members of the expanded EXO70 gene family. Interestingly, a number of the EXO70 members are linked to cellular responses during plant–microbe interactions and defences.

While the conventional secretory pathway produces secretory vesicles for transport to the plasma membrane, there are pathways that involve multivesicular bodies (MVBs) instead. MVBs carry intralumenal vesicles which are then released upon fusion with the plasma membrane (termed extracellular vesicles or exosomes: reviewed by Goring, 2017; Hansen and Nielsen, 2017; Wang *et al.*, 2017). One of the early examples of this mechanism in plants was documented in barley cells where MVBs were observed releasing exosomes to thwart the invading powdery mildew fungus (An *et al.*, 2006). Hansen and Nielsen (2017) review the trafficking pathways that regulate this response where plant cells release exosomes during two phases of powdery mildew infection: pre-invasive immunity (papilla formation) and post-invasive defence (encasement formation). Very little is known about biogenesis of MVBs for exosome secretion, and in studying potential trafficking components, the authors have unexpectedly discovered that encasement formation appears to have linkages to cell plate formation and is regulated by a different trafficking pathway to papilla formation (Hansen and Nielsen, 2017; Nielsen *et al.*, 2017). Exosome secretion has also been documented with compatible pollinations in *Brassica* species (reviewed by Goring, 2017) which is quite interesting as parallels are often drawn between plant–fungal and pollen–pistil interactions (Chen *et al.*, 2015).

Determining the cargo of secreted extracellular vesicles in plants is an important step forward in understanding their functions as described by two recent proteomic studies. Rutter and Innes (2017) collected Arabidopsis leaf apoplastic fluids while Regente *et al.* (2017) used extracellular fluids from sunflower seedlings to extract extracellular vesicles for proteomic analyses. Both found an enrichment in plant defence-related protein compounds and, interestingly, Regente *et al.* (2017) also found that these extracellular vesicles could be taken up by the sunflower fungal pathogen *Sclerotinia sclerotiorum* (see also the Insight article by Boevink, 2017). To fully decipher the roles and mechanisms of these novel trafficking pathways, the integration of new technologies and approaches will be important. Along these lines, Wang *et al.* (2017) explain the use of advanced microscopy systems while Rodriguez-Furlan *et al.* (2017) discuss the use of small molecule inhibitors to dissect the trafficking routes being taken.

## Unconventional vacuolar traffic will soon become conventional…

Vacuolar sorting mechanisms are often identified with the trafficking of vacuolar sorting receptors, but the traffic of an increasing number of vacuolar proteins, in particular membrane proteins, appears independent. Three reviews in this issue touch on this aspect of vacuolar trafficking, but coming from different perspectives (Bellucci *et al.*, 2017; Di Sansebastiano *et al.*, 2017; Pecenkova *et al.*, 2017). It is evident from these reviews that the vacuolar trafficking pathways are more complex and interconnected than previously thought. Since vacuoles receive cargo molecules and membranes that also define the functional specificity of the different donors, this so-called ‘unconventional traffic’ will ultimately affect cell compartmentalization. The ER is the most important donor to the vacuole, but the plasma membrane and Golgi apparatus are also donors. Thus, understanding the ‘unconventional trafficking’ pathways will be essential to fully interpret how these intracellular processes function.

Di Sansebastiano *et al.* (2017) review the classical route followed by vacuolar sorting receptors and then several alternative routes. Some alternative sorting pathways such as for AP-3 and dense vesicles require the Golgi apparatus, but others appear to be Golgi-independent. Direct ER-to-vacuole pathways appear to be linked to autophagy-related processes, and the trafficking of metabolites such as anthocyanins provide an interesting example of this (reviewed by Pecenkova *et al.*, 2017). Other vacuolar proteins such as Chitinase A (Stigliano *et al.*, 2013), Cardosines (Pereira *et al.*, 2013) and several membrane proteins [Occhialini *et al.*, 2016; Pedrazzini *et al.*, 2016 (see also the Insight article by Strasser, 2016); Pompa *et al.*, 2017] are examples that do not follow the conventional vacuolar trafficking pathway, but where the mechanisms are unclear. Finally, the mechanisms regulating direct routes of transport from the cytosol to vacuolar membrane (tonoplast) also need to be explored in more depth. Nevertheless, a clear message is that Golgi-independent vacuolar trafficking is not exceptional, but a fundamental process that is still poorly understood and underestimated (Di Sansebastiano *et al.*, 2017).

## New models for cell-to-cell traffic through plasmodesmata

The trafficking events described above have focused on the movement of cargo between different organelles within the plant cell, but movement of proteins and metabolites can also occur between cells through plasmodesmata. These intercellular channels not only connect the cytosols of neighbouring cells, but their plasma membrane and ER are interconnected across these channels. Nicolas *et al.* (2017*a*) review the dynamic structure of these closely packed, plasmodesma-associated membranes. The lumen of the crossing ER strand (‘desmotubule’) appears to be tightly compressed, which raises the question as to whether lumenal transport can actually occur across the plasmodesmata and, if not, then the actual function of the desmotubule. Accordingly, most of the plasmodesmal trafficking is thought to occur in the gap between the plasma membrane and the desmotubule, termed the cytoplasmic sleeve. The width of this gap can vary during development and appears to be influenced by ER–plasma membrane contacts that bring the desmotubule and plasma membrane very close together in young plasmodesmata, but are more relaxed when they have matured (Nicolas *et al.*, 2017b). Ultimately, these structure–function studies have important implications for understanding how plasmodesmal permeability (i.e. size exclusion limit) is impacted by the width of the cytoplasmic sleeve and the ER–plasma membrane contact sites. Nicolas *et al.* (2017*a*) also discuss the notion of membrane microdomains, which have distinct protein and lipid compositions, and how they define specialized functions for plasmodesmata.

An additional level of regulation for plasmodesmal transport capacity is through modifications of the (1,3)-β-glucan polymer, callose, in the cell wall flanking the plasmodesmata. Amsbury *et al.* (2017) review the signalling pathways that regulate callose turnover through the regulation of enzymes for its synthesis and degradation. The accumulation of callose in the cell wall forms a collar around the plasmodesma to decrease the aperture size, and this is turn would reduce transport flux. They propose a model where the general cell wall structure would set the mechanical limit for the transport of macromolecules, but the regulation of callose synthesis/degradation would allow for dynamic changes within these limits. Finally, Amsbury *et al.* (2017) discuss other cell wall components, such as the pectin network, that could participate in the regulation of plasmodesmal permeability. How callose and these other cell wall components are coordinately regulated to control transport through plasmodesmata will also need to be deciphered.

Plant viruses take advantage of the transport capacity of plasmodesmata to spread from cell to cell. Pitzalis and Heinlein (2017) review the literature on how different plant viruses use the plasmodesma-associated membranes and cytoskeleton for viral movement, and offer new interpretations on the proposed mechanisms. Plant viruses need to increase the size exclusion limit of the plasmodesmata for crossing, and can increase the channel diameter by inducing callose-degrading glucanases (Zavaliev *et al.*, 2013). However, viral movement proteins also increase size exclusion limits, and the mechanism of movement protein action is the topic under discussion by Pitzalis and Heinlein (2017). A few plant viruses require the conventional ER–Golgi secretory pathway to propagate and spread while many other plant viruses only use the ER membrane without the involvement of the Golgi. In the latter instance, Pitzalis and Heinlein (2017) surmise that rather than using the ER membrane system to move across the plasmodesmata, the viruses are actually using the ER-associated actin network and myosin motor proteins for movement. This actin–myosin system provides directional transport in the cell, and most plant viruses probably depend on myosin XI for general cell motility (Amari *et al.*, 2014; Griffing *et al.*, 2017). However, Pitzalis and Heinlein (2017) propose that for viruses using the ER actin–myosin system, myosin VIII is important for manipulating the plasmodesmal permeability to cross into neighbouring cells. As discussed by Nicolas *et al.* (2017*a*) the ER–plasma membrane contact sites can result in tight interactions between these two membranes across the plasmodesmata. Pitzalis and Heinlein (2017) describe a model where myosin VIII interacts and regulates the length of a plasmodesma-localized tethering protein at the ER–plasma membrane contact site. The viral movement proteins are then proposed to interfere with the myosin VIII-tethering protein interaction, and allow for the expansion of tethering protein to favour viral RNA traffic through the expanded channel.

## Conclusions

As summarized in the Box 1 model, there are many different routes that can be used to move cargo from one compartment to the next in the cell, and a central question is why these different routes exist. Some of these examples, such as the exosomes, are linked to pathogen defence responses and perhaps have evolved as an effective method to round up and deliver the defence-related cargo. Other examples are linked to specialized structures, with variations in how the proteins are trafficked. For example, the movement of storage proteins to protein storage vacuoles in seeds can take different routes such as the conventional ER–Golgi–vacuole, direct ER–vacuole, and the incorporation of autophagy-related structures as intermediates in the ER–vacuole pathway. Finally, there are already hints that as some of these ‘unconventional’ pathways become better understood, they have the potential to be properly seen as the more common trafficking routes used in the plant cell. Accordingly, not so ‘unconventional’ after all.

**Box 1. The many trafficking routes that cargo can take in the plant cell**

The conventional secretory pathway starts at the endoplasmic reticulum (ER), with proteins destined for the plasma membrane translocated into this complex organelle during synthesis. From the ER, these proteins are exported to the Golgi and then delivered in secretory vesicles to the plasma membrane, either directly from the Golgi (2) or first routing through the *trans*-Golgi network (TGN) (3). Unconventional routes include direct ER–plasma membrane trafficking (1), and the use of multivesicular bodies to secrete extracellular vesicles/exosomes (4). There are also cytosolic leaderless secreted proteins (LSPs) that lack ER sorting signals and somehow are delivered to the apoplast from the cytoplasm; these proteins have been proposed to be secreted through exocyst-positive organelles (EXPOs, labelled ‘E’) (5). Other unconventional routes to the plasma membrane include direct trafficking from the vacuole to the plasma membrane (6), and a potential link between autophagy and plasma membrane-destined multivesicular bodies (7).

The second direction of trafficking shown in this model is to the vacuole. The conventional route is from the TGN (early endosome) to the prevacuolar compartment (late endosome/multivesicular body), and onto the vacuole (8). Unconventional delivery of cargo to the vacuole includes direct trafficking routes from the Golgi (9) or the ER (10), as well as autophagy-linked routes from the ER (11) or the cytoplasm (12).

The last direction of trafficking is the movement of proteins, RNA and metabolites between plant cells through the plasmodesmata (PD) (13). Plant viruses also exploit plasmodesmata to spread from cell to cell.

**Figure:**